# Immediate Postoperative Complications in Adult Tracheostomy

**DOI:** 10.7759/cureus.12228

**Published:** 2020-12-22

**Authors:** Samba Siva Bathula, Luxman Srikantha, Tyler Patrick, Noah A Stern

**Affiliations:** 1 Otolaryngology, Michigan State University Detroit Medical Center, Detroit, USA

**Keywords:** tracheostomy, tracheostomy complications, air way complications

## Abstract

Objective

Tracheostomy is one of the oldest operations for the management of airway obstruction. With time, indications expanded to prolonged mechanical ventilation, and currently, the majority of tracheostomies are done for this reason. There are several techniques used in a tracheostomy procedure, depending on surgeon preference. Immediate complications such as bleeding, pneumothorax, pneumomediastinum, airway fire, and posterior tracheal wall perforation with esophageal injury are rare, although they do occur, and must be managed accordingly. This study aimed to assess differences in types and rates of immediate postoperative complications in patients undergoing tracheostomy when performed under general anesthesia and local anesthesia (awake tracheostomies) at a large academic institution. This is a continuing ongoing literature reporting tracheostomy adverse events.

Methods

A retrospective chart review was performed to identify patients who underwent tracheostomy placement between January 1, 2013 and December 31, 2019 at the Detroit Medical Center, USA. Postoperative complications such as bleeding, pneumothorax, pneumomediastinum, airway fire, and posterior tracheal perforation were collected along with gender, age, and revision tracheostomy status. IBM SPSS Statistics (IBM Inc., Armonk, USA) was used for statistical analysis with the statical significance defined as a p<0.05.

Results

A total of 1,469 patient charts were reviewed. Of these, 1,342 met the inclusion and exclusion criteria, of which, males were 57.2% (n=768), and females were 42.8% (n=574). The age range was 18 years to 96 years (mean=58.03; SD= 15.97), and BMI range was 12-83 (mean=28.77; SD=7.885).

Multinomial logistic regression was performed to determine whether age, BMI, sex, and revision tracheostomies were represented across both general and awake tracheostomy groups proportionally to their numbers in the total sample. It showed non-significant value for age (χ2=0.776, p=0.378), BMI (χ2=0.004, p=0.947), but significant value for sex (χ2=4.645, p=0.031), revision tracheostomy (χ2=18.282, p<0.001), indicating that males and revision tracheostomies over-represented in awake tracheostomies.

Next, Pearson correlation analysis was performed to determine any significant linear relationship between age, sex, and tracheostomy complications. It showed a significant positive correlation between age and tracheal stomal infection [r(1,340)=0.062, p=0.022].

An independent sample t-test showed a statistically significant difference between the mean pneumothorax and pneumomediastinum of general (n=1,277, mean=0.01, SD=0.088) and awake tracheostomies (n=65, mean=0.08, SD=0.269, t=2.069, p=0.043).

Pneumothorax pneumomediastinum complications between the general tracheostomy and awake tracheostomy odds ratio (OR)-6.22, indicates the chance of pneumothorax /pneumomediastinum complication is 6.22 times more in awake tracheostomy than general tracheostomy. Based on the above statistical analysis, we rejected the null hypothesis.

Conclusions

Tracheostomy is the procedure of choice to relieve the upper airway obstruction and treat patients requiring prolonged mechanical ventilation. A slightly higher number of Immediate postoperative complications in awake tracheostomy were noticed in patients with more surgically challenging revision tracheostomies.

## Introduction

Tracheostomy is one of the oldest operations for the management of airway obstruction. Depictions of tracheostomy are visible on Egyptian tablets from 3600 BCE [1]. Tracheostomy surgery was widely used during the polio epidemic to relieve upper airway obstruction after introducing a safer standardized technique, introduced by Chevalier Jackson in the 20th century [2]. Later, tracheostomy surgeries were also indicated for prolonged mechanical ventilation, and currently, the majority of tracheostomies are done for ventilator-dependent respiratory failure. The American College of Chest Physicians recommends considering a tracheostomy for patients who require an endotracheal tube for more than 21 days [3]. Benefits of establishing a tracheostomy rather than using an endotracheal tube include decreasing direct laryngeal injury, improving comfort, and improving daily living activities such as mobility, speech, eating, and lower intensive care unit (ICU) and in-hospital length of stays [4-6]. 

The average number of tracheostomies performed annually in the United States at present is greater than 100,000 [3]. This procedure is commonly performed by general surgeons and otolaryngologists. There are several techniques used in a tracheostomy procedure, depending on surgeon preference [7]. Immediate complications such as bleeding, pneumothorax, pneumomediastinum, airway fire, and posterior tracheal wall perforation with esophageal injury are rare, although they do occur and must be managed accordingly [8, 9]. 

This study aims to assess differences in types and rates of immediate postoperative complications in patients undergoing tracheostomy when performed under general anesthesia (general tracheostomies) and local anesthesia (awake tracheostomies) at a large academic institution. This is a continuing ongoing literature reporting tracheostomy adverse events. Our null hypothesis is that there will be no significant differences in immediate postoperative tracheostomy complications between general tracheostomies and awake tracheostomies.

## Materials and methods

A retrospective research methodology was employed for this investigation. Institutional Review Board approval was obtained. Inpatient medical charts from the Detroit Medical Center, USA, were then examined in detail to identify adults who had undergone a tracheostomy procedure between January 1, 2013 and December 31, 2019. Inclusion criteria include all open tracheostomies that were performed in the Detroit Medical Center on patients above the age of 18. Exclusion criteria included percutaneous tracheostomy, conversion tracheostomies from prior cricothyroidotomies.

The operative report of every patient was scrutinized to confirm the details of an open tracheostomy. With some variation in techniques, the surgical procedure was as follows: the patient was kept in a supine position, and a shoulder roll was placed underneath the shoulder. The skin incision was given over the anterior neck. Strap muscles were separated in the midline, and the thyroid isthmus was divided to expose the second and third tracheal rings. The fraction of inspired oxygen (FI02) was asked to be less than 30% prior to opening the trachea on a patient with ventilation under general anesthesia. The tracheal opening was done between the second and third tracheal rings with a knife. A tracheostomy tube with an obturator was introduced into the trachea lumen and gently inserted into the trachea without excessive force to prevent posterior tracheal wall injury. Depending on body habitus, the size of the pre-existing endotracheal tube, either a size 6.0 or 8.0 Shirley™ cuffed tracheostomy tube, was inserted after opening the trachea, and anesthesia circuitry was connected. End-tidal carbon dioxide (CO2) was confirmed. The tracheostomy tube was fixed in situ by suturing the flanges to neck skin with 2-0 Prolene or silk on each side. Tracheostomy was further secured with a Posey collar. Tracheostomy flange sutures were removed anywhere from the third to the seventh postoperative day, depending on the surgeon's preference. Proximal XLT tracheostomies were most often used for those patients with BMI >35 (class-2 obesity) and distal XLT tracheostomy tubes were often used to bypass a distal area of tracheal stenosis. Occasionally uncuffed tracheostomy tubes were used whenever unable to pass a cuffed tracheostomy tube due to tracheal stenosis. Custom made tracheostomy tubes were rarely used for morbidly obese patients. Prior to the procedure, antiplatelets and anticoagulants such as aspirin, clopidogrel, and warfarin were held at least five days prior to the procedure except for emergent awake tracheostomies. Enoxaparin and heparin were discontinued at least eight hours prior to the procedure. For ventilator-dependent patients, positive end-expiratory pressure (PEEP) was at most 8 cm H2O, and the fraction of inspired oxygen (FIO2) was 60% or less. We did not use any sedative medications within our institution before awake tracheostomies because this reduces respiratory drive in an already compromised airway.

SPSS (IBM Inc., Armonk, USA) power analysis was done to estimate the sample size for independent means with a 99% confidence level and 1% margin of error. The minimum requirement was 64 subjects in each group. The charts of more than 1,400 subjects were ultimately examined.

For the purpose of interrater reliability, all three judges re-reviewed (two weeks later) 50% of the charts they originally analyzed for comparative analyses with their original findings; 100% agreement on all original and subsequent chart data extractions was required for this reliability measure. Discrepancies were resolved by multiple re-reviews to ensure complete accuracy of all chart extractions. Selection bias was reduced using the above strict review process. None of the reviewers were aware of any complications of general tracheostomies and awake tracheostomies prior to reviewing.

SPSS Statistics was used for descriptive statistical analysis, including chi-square and the Fisher exact test calculations for comparisons among the categorical variables, Pearson r-correlation, and independent T-test to compare the continuous variables with the statical significance of p<0.05.

## Results

The purpose of this retrospective study was to determine the differences in types and rates of immediate postoperative complications in patients undergoing general and awake tracheostomies in the first seven postoperative days. The patients were noted to be of the same pre-operative risk as more than 95% of the patients' preoperative American Society of Anesthesiologist Score was three or above.

The examined dependent variables were post-operative bleeding, pneumothorax, pneumomediastinum, posterior tracheal wall perforation, and tracheal stomal infection. The independent variables were the two general and awake tracheostomies. Additionally, three covariates (gender, age, and BMI) and revision tracheostomies were also analyzed.

After controlling for the covariates, our null hypothesis is that there will be no significant differences in immediate postoperative tracheostomy complications (bleeding, pneumothorax, pneumomediastinum, airway fire, or posterior tracheal perforation) between general tracheostomies and awake tracheostomies. A p-value of more than 0.05 indicates no significant difference between the general tracheostomies and awake tracheostomies.

**Figure 1:**
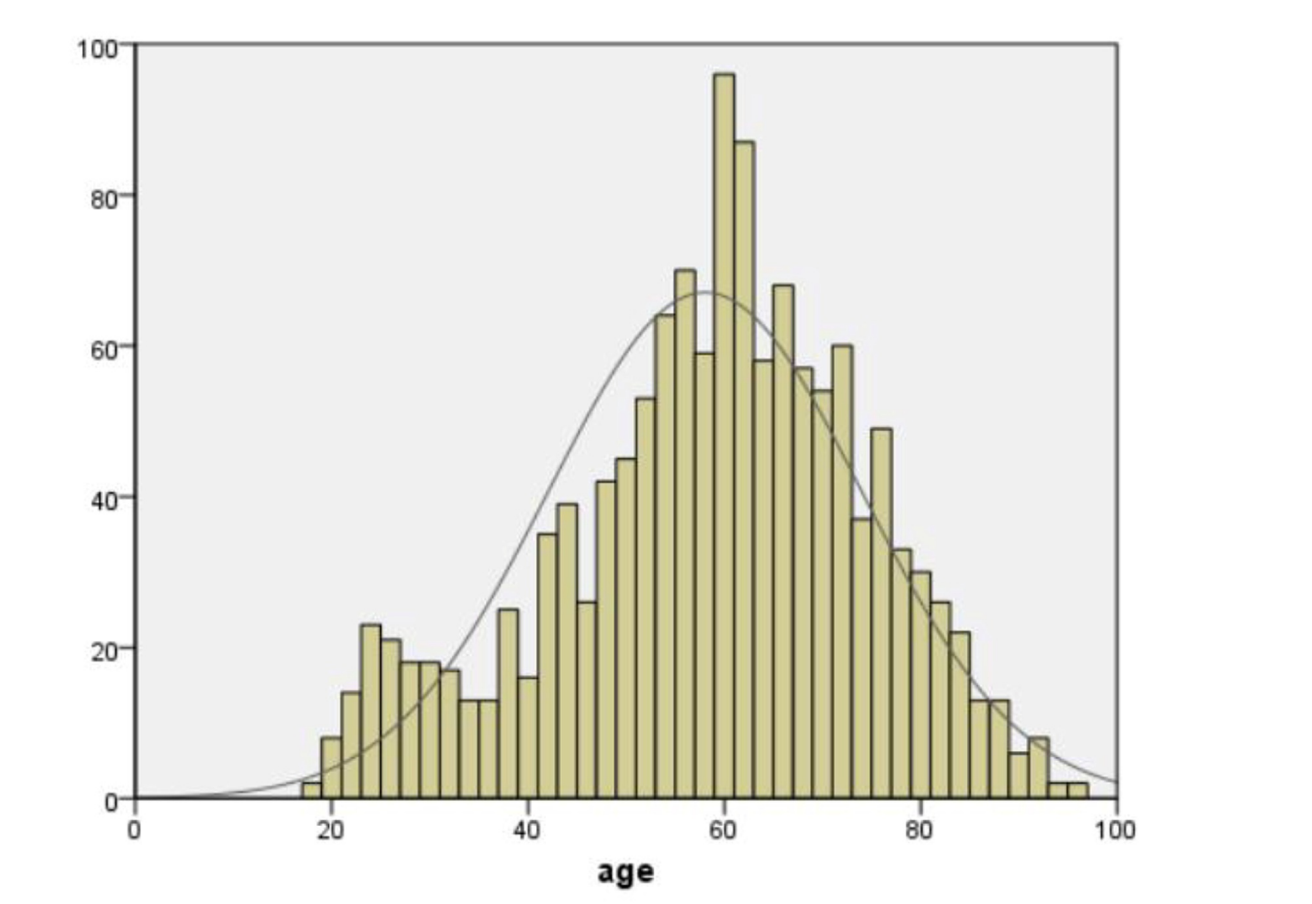
Age distribution histogram in tracheostomy

**Figure 2:**
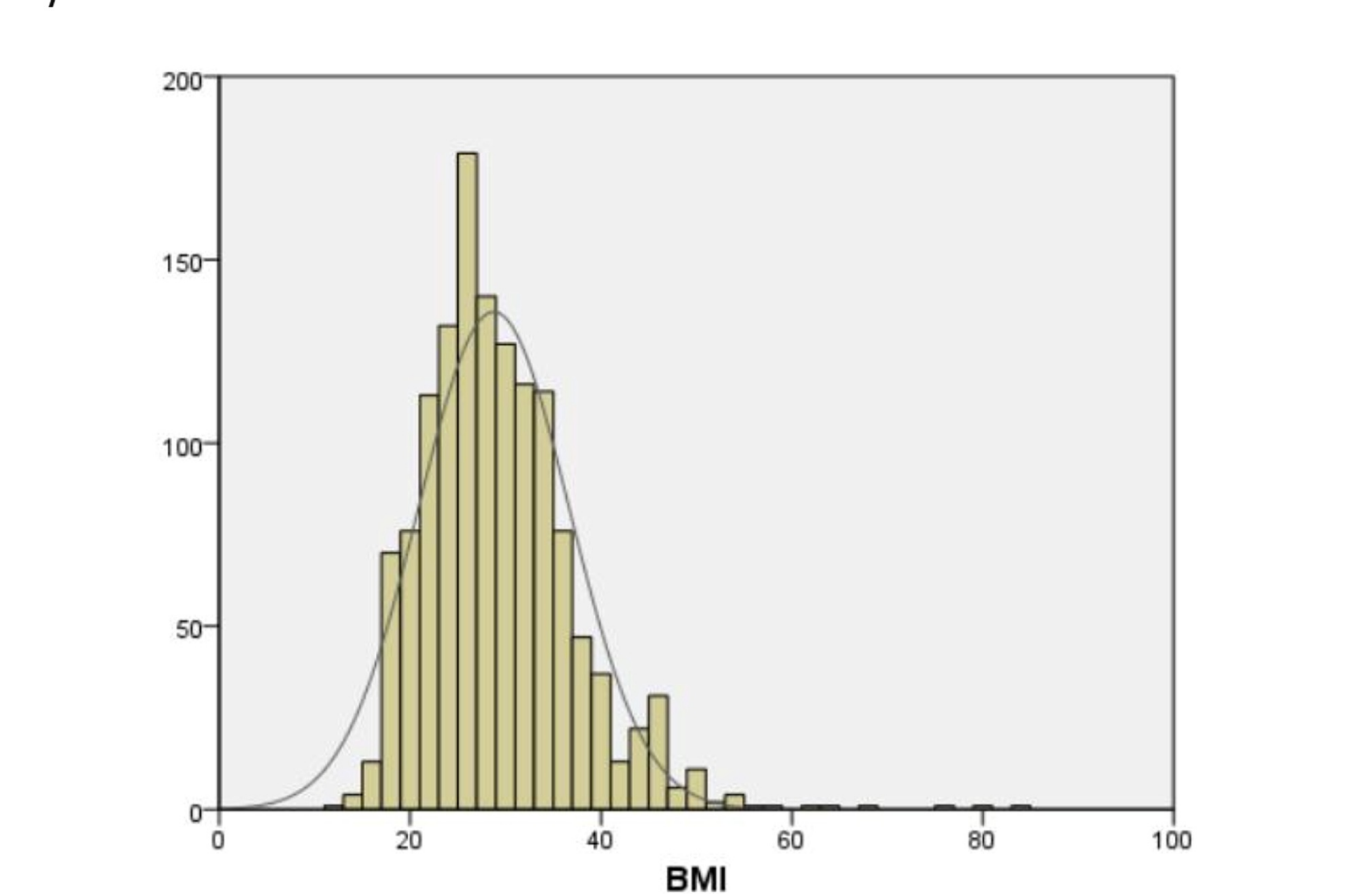
Body mass index (BMI) distribution histogram in tracheostomy

A total of 1,469 patient charts were reviewed. Out of these, 1,342 met the inclusion and exclusion criteria, of which 811 were performed by general surgery physicians and 531 by otolaryngology physicians. There were 57.2% (n=768) males and 42.8% (n=574) females. According to the age distribution histogram (see Figure 1), the age range was 18 to 96 years (mean=58.03; SD= 15.97). The BMI range was 12 to 83 (mean=28.77; SD=7.88), as shown in Figure 2.

Multinomial logistic regression was performed to determine whether age, BMI, sex (dummy codes: male=1, female=0), and revision tracheostomies were represented across both general and awake tracheostomy groups (dummy codes: general=1, awake=0) proportionally to their numbers in the total sample. This measure produced a non-significant value for age (χ2=0.776, p=0.378), BMI (χ2=0.004, p=0.947), indicating that age range and BMI range were not over-represented in any of the two categories (general tracheostomies and awake tracheostomies). But significant value for sex (χ2=4.645, p=0.031), revision tracheostomy (χ2=18.282, p<0.001) indicating that males and revision tracheostomies were over-represented in awake tracheostomies (Table 1). Male sex and revision tracheostomies had a negative correlation what means that these patients are less likely to undergo general tracheostomies than awake tracheostomies. Male sex odds ratio (OR=1.8) indicates that male patients are 1.8 times more likely to get awake tracheostomy than general tracheostomy. Revision tracheostomy odds ratio (OR=5.2) indicates that revision tracheostomy patients are 5.2 times more likely to get awake tracheostomy than general tracheostomy.

**Table 1:** Multinomial logistic regression: age, BMI, sex and revision tracheostomies distribution in general and awake tracheostomy groups BMI - body mass index

Effect	Chi-square	p
Age	0.776	0.378
Sex (dummy codes: male=1, female=0)	4.645	0.031
BMI	0.004	0.947
Revision tracheostomy	18.282	<0.001

Pearson correlation analysis was performed to determine any significant linear relationship between the age and tracheostomy complications (see Table 2). No significant relationship with tracheostomy bleeding [r(1,340)=0.048, p=0.081], pneumothorax/pneumomediastinum [r(1,340)=-0.027, p=0.326] and posterior tracheal wall perforation [r(1,340)=0.010, p=0.709] were noted. But there was a significant positive correlation with tracheal stomal infection [r(1,340)=0.062, p=0.022].

**Table 2:** Pearson correlation chart *Correlation is significant at the 0.05 level (2-tailed).

		Trach bleeding	Pneumothorax/ pneumomediastinum	Tracheal stoma infection	Posterior tracheal wall perforation
Age	Mean	0.03	0.01	0.01	0
	Standard deviation	0.168	0.105	0.09	0.027
	Pearson correlation	0.048	-0.027	0.062*	0.01
	Sig. (2-tailed)	0.081	0.326	0.022	0.709
BMI	Mean	0.03	0.01	0.01	0
	Standard deviation	0.168	0.105	0.09	0.027
	Pearson correlation	0.028	0.014	0.009	-0.01
	Sig. (2-tailed)	0.313	0.611	0.743	0.726
	N	1,342	1,342	1,342	1,342

Next, Pearson correlation analysis was performed to determine any significant relationship between the BMI and tracheostomy complications. No significant relationship with tracheostomy bleeding [r(1,340)=0.028, p=0.313], pneumothorax/pneumomediastinum [r(1,340)=0.014, p=0.611], tracheal stomal infection [r(1,340)=0.009, p=0.743] and posterior tracheal wall perforation [r(1,340)=-0.010, p=0.726] was noted.

Chi-square analysis was conducted to determine whether males and females in the entire study population significantly differed in the number of tracheostomy bleeding and pneumothorax/pneumomediastinum. The distribution of the data set produced non-significant Chi-square values for tracheostomy bleeding(χ2=0.045, p=0.833 ) and pneumothorax/pneumomediastinum (χ2=0.000, p=0.989 ).

In terms of tracheostomy indications, malignancy was the primary indication for awake tracheostomy (48/61, 78.7%), whereas ventilatory dependent respiratory failure (VDRF)** **was for general tracheostomy (1238/1277, 96.9%). Other indicators were laryngeal trauma, bilateral vocal cord paralysis, angioedema, laryngeal and tracheal stenosis. Patients with upper airway obstruction had 2.56 times to undergo awake tracheostomy than general tracheostomy (OR=2.56, p<0.001).

An independent sample t-test was conducted to determine if there is a difference between the mean tracheostomy complications such as tracheostomy bleeding, pneumomediastinum and pneumothorax, tracheal stomal infection, and posterior tracheal wall perforation of general and awake tracheostomies (Table 3).

**Table 3:** Independent sample t-test: mean tracheostomy complications in general and awake tracheostomies

	General tracheostomy-1, awake tracheostomy-0	N	Mean	Std. deviation	Std. error mean	t	Sig. (2-tailed)
Trach bleeding	1	1277	0.03	0.168	0.005	-0.084	0.933
	0	65	0.03	0.174	0.022		
Pneumothorax/pneumomediastinum	1	1277	0.01	0.088	0.002	-2.069	0.043
	0	65	0.08	0.269	0.033		
Tracheal stoma infection	1	1277	0.01	0.092	0.003	0.751	0.453
	0	65	0	0	0		
Posterior tracheal wall perforation	1	1277	0	0.028	0.001	0.226	0.822
	0	65	0	0	0		

There was no statistically significant difference between mean tracheostomy bleeding complications of general (n=1277, mean=0.03, SD=0.168) and awake tracheostomies (n=65, mean=0.03, SD=0.174, t=0.084, p=0.933), mean tracheal stomal infection of general (n=1277, mean=0.01, SD=0.092) and awake tracheostomies (n=65, mean=0.00, SD=0.00, t=0.751, p=0.453), mean posterior tracheal perforation of general (n=1277, mean=0.00, SD=0.028) and awake tracheostomies (n=65, mean=0.00, SD=0.00, t=0.226, p=0.822). But there was a statistically significant difference between the mean pneumothorax and pneumomediastinum of general (n=1277, mean=0.01, SD=0.088) and awake tracheostomies (n=65, mean=0.08, SD=0.269, t=2.069, p=0.043).

Pneumothorax pneumomediastinum complications between the general tracheostomy and awake tracheostomy odds ratio (OR)=6.22 indicates that the chance of pneumothorax /pneumomediastinum complication is 6.22 times more in awake tracheostomy than in general tracheostomy. Based on the above statistical analysis, the null hypothesis was rejected.

There was one case of airway fire in the general tracheostomy group. No tracheostomy surgery-related deaths were noted in the operating room in either group.

## Discussion

Tracheostomy is a common surgical procedure performed on prolonged ventilation dependent patients in the intensive care unit or to treat individuals with impending upper airway obstruction due to infection, inflammation, maxilla-facial trauma and edema, and head and neck cancer. The majority of tracheostomies in our study were done under general anesthesia with pre-existing endotracheal intubation, whereas awake tracheostomy is usually performed for patients with severe upper airway obstruction, causing unable to intubate [10]. 

According to Doron Sagiv et al., awake tracheostomies had a slightly higher risk of immediate postoperative complications [11]. In our study, the awake tracheostomy group had slightly more immediate postoperative complications such as pneumothorax, pneumomediastinum, and subcutaneous emphysema complications, which would be expected with the greater number of revision and awake tracheostomies performed. These surgeries tend to be more complicated due to the higher acuity of the patient's needs and the difficulty of the procedure [12-15]. Furthermore, two of them were undergoing a revision awake tracheostomy. Lastly, the posterior tracheal wall perforation occurred on a patient having his fourth revision awake tracheostomy. The revision was performed awake due to tracheal stenosis.

The most common cause for revision tracheostomy was required in prolonged mechanical ventilation after tracheostomy decannulation (n=63/76, 82%). Four cases of third tracheal ring stenosis after tracheostomy decannulation was noted. Nine cases of accidental tracheostomy decannulation and complete closure of tracheal stoma was noted before tracheostomy tube reinsertion.

Postoperative tracheostomy bleeding was around 5% in the literature, but only 2.9% in our study [16]. The causes of tracheostomy bleeding in our study (39 patients) were from anterior jugular veins, thyroid isthmus, history of anticoagulant medications, and minor mucosal bleeding around the tracheal stoma by unknown cause similar to that noted in the literature [17]. Sixty-nine percent (27 out of 39) of tracheostomy bleeding was minor mucosal bleeding around the tracheal stoma and was resolved with the surgical application at the bedside. Bleeding was caused due to the anterior jugular vein in 10.2% (4/39) patients and the thyroid isthmus in 12.8% (5/39) patients; these were controlled by neck exploration under general anesthesia. One case of innominate artery injury during awake revision emergency tracheostomy was encountered in a patient with a high riding innominate artery and was successfully repaired at the same time without significant morbidity to the patient. Five percent (2/39) of patients were on active anticoagulant medications or antiplatelet medications at the time awake emergency tracheostomy - bleeding episodes were controlled with tracheal stoma packing with surgical ribbon gauze packing, blood transfusion, and immediate discontinuation of anticoagulant medication or antiplatelet medications. Both patients’ bleeding was controlled within 72 hours. No deaths were noted due to excessive bleeding following tracheostomy in our study group.

In our study, only 14.3% (2/14) cases of pneumothorax and/or pneumomediastinum occurred during tracheostomy, and both required chest tubes. Another 85.7% (12/14) cases of pneumomediastinum and/or neck subcutaneous air were resolved with stab incision “blow-holes” in the neck.

Only one case of airway fire was noted in our study. It happened when the surgeon accidentally entered the tracheal lumen while dividing the thyroid isthmus with electric cautery. The airway fire was controlled immediately by simultaneous removal of cautery from fire, saline irrigation, and removal of the endotracheal tube [18]. Intra-operative bronchoscopy showed tracheal wall burn injury at the level of the second and third tracheal rings. The remainder of the tracheobronchial wall appeared normal. The patient was monitored first for the 10 days postoperatively and noted delayed stomal wound healing.

Although this study does not support the null hypothesis, there is a relatively low number of complications from both general and awake tracheostomies compared to numbers available in the literature [19].

Limitations of this study relate to its retrospective nature and the fact that it was conducted at a single academic institution. Additionally, although the same surgical steps were acknowledged when reviewing the operative reports, some surgeons employed slightly varying surgical techniques, which were difficult to control due to the study's retrospective nature.

## Conclusions

Tracheostomy is the procedure of choice to relieve the upper airway obstruction and treat patients requiring prolonged mechanical ventilation. A slightly higher number of immediate postoperative complications in awake tracheostomy were noticed in patients with more surgically challenging revision tracheostomies.
